# Report on the 6th scientific meeting of the “Verein zur Förderung des Wissenschaftlichen Nachwuchses in der Neurologie” (NEUROWIND e.V.) held in Motzen, Germany, Oct. 31th – Nov. 2nd, 2014

**DOI:** 10.1186/s13231-014-0013-z

**Published:** 2015-01-13

**Authors:** Christoph Kleinschnitz, Ralf A Linker, Tim Magnus, Thomas Korn, Sven G Meuth, on behalf of the speakers at the 6’th NEUROWIND e.V. scientific meeting

**Affiliations:** Department of Neurology, University Clinic of Würzburg, Josef-Schneider-Str. 11, 97080 Würzburg, Germany; Department of Neurology, Friedrich-Alexander-Universität Erlangen, Schwabachanlage 6, 91054 Erlangen, Germany; Department of Neurology, University Clinic Hamburg-Eppendorf, Martinistr. 52, 20246 Hamburg, Germany; Department of Neurology, Klinikum rechts der Isar, Technische Universität München and Munich Cluster for Systems, Neurology (SyNergy), Ismaninger Str. 22, 81675 Munich, Germany; Department of Neurology, University of Münster, Albert-Schweitzer Campus 1, 48149 Münster, Germany

## Abstract

From October 31th – November 2nd, 2014, the 6th NEUROWIND e.V. meeting was held in Motzen, Brandenburg, Germany. 70 doctoral students and postdocs from over 25 different groups working in German and Swiss university hospitals or research institutes attended the meeting to discuss their latest experiments and findings in the fields of neuroimmunology, neurodegeneration and neurovascular research. The meeting was regarded as a very well organized platform to support research of young investigators in Germany and all participants enjoyed the stimulating environment for lively in depth discussions.

According to the major aim of NEUROWIND e.V. to support younger researchers in Germany the 4th NEUROWIND YOUNG SCIENTIST AWARD for experimental neurology was awarded to Michael Breckwoldt on his work in the group of Thomas Misgeld (Institute of Neuronal Cell Biology, Technische Universität München, Germany). The successful project was published in *Nature Medicine* entitled “Multiparametric optical analysis of mitochondrial redox signals during neuronal physiology and pathology *in vivo*”. This outstanding paper deals with a molecular imaging approach in living mice to optically analyze the role of mitochondrial redox signals in axons in health and disease. The award is endowed with 20.000 Euro sponsored by Merck Serono GmbH, Darmstadt, Germany (unrestricted educational grant).

This year’s keynote lecture was given by Bernhard Hemmer, Head of the Department of Neurology at the Klinikum rechts der Isar, Technische Universität München. Dr. Hemmer highlighted the particular role of B cells and (auto)antibodies in multiple sclerosis (MS). As a new highlight Dr. Urbahns, head of global discovery technologies at Merck research laboratories, gave insights from research practice in the pharmaceutical industry and introduced a shift in the view on present-day drug discovery paradigms.

## Summary of the scientific contributions to the NEUROWIND e.V. meeting 2014

### Contributions on vascular neurology

Kathrin Schüller from Nikolaus Plesnila’s group in Munich focused on a mouse model of subarachnoid hemorrhage demonstrating early microcirculatory dysfunction (e.g. changes in vessel diameter, blood flow velocity). Next, Robert Brunkhorst from the group of Waltraud Pfeilschifter, Frankfurt, gave a talk on neuroregenerative effects of selective serotonin reuptake inhibitors (SSRI) and the sphingolipid-metabolism after induction of photothrombotic stroke. Arthur Liesz from Munich studied the release of alarmins also called DAMPs (danger-associated molecular pattern) following MCAO. In mice and men a stroke-size dependent up-regulation of high-motility group protein 1 (HMGB1) could be shown, although it has to be taken into account that signals differ in the subacute and the chronic phases after stroke induction. Christoph Harms from Berlin discussed pros and cons of preclinical stroke models and highlighted potential roadblocks in translational stroke research. He pointed out that SUMO2/3 (small ubiquitin-like modifier proteins) and their regulation by SENP7 (sentrin specific peptidase 7) might contribute to endogenous neuroprotection.

Hagen Huttner from Erlangen reported on the radiocarbon method as an innovative strategy to investigate adult neurogenesis in the human brain, e.g. in stroke. The data clearly show that after cortical infarction, there is no generation of new neurons in the brain. Kai Diederich from the group of Jens Minnerup, Münster, studied the role of lymphocytes for regeneration in a long-term photothrombosis model of cerebral ischemia. Thorsten Doeppner from Dirk Herrmann’s group in Essen critically discussed the value of neuronal stem cells and exosomes as neuroregenerative approach in cerebral ischemia. Finally, Stine Mencl from Christoph Kleinschnitz’s group reported on paradigms of neuroprotection in stroke models using NMDAR activated NOS molecules as targets of potential neuroprotective approaches. Blocking the NOS pathway and thus reducing toxic NO production appeared promising for protecting neuronal tissue after stroke. Three inhibitors Tat-NR2B9c, Tat-N-Dimer, and ZL006 were tested in the transient and permanent MCAO paradigm in mice and rats. In contrast to what was reported in the literature, no beneficial effect of any of the NOS pathway inhibiting compounds was detected. This reinvigorates the upcoming discussion on the role of preclinical stroke models for testing neuroprotective compounds to be used in human stroke patients.

### Neuroimmunology and oligodendroglial biology

Johannes Herkel from Hamburg gave a talk on tolerance-inducing liver cells controlling distinct stages of inflammation, e.g. in EAE animals. His group used nanoparticles as carrier structures to deliver antigens to liver endothelial cells. The concept is translated to humans by initiation of a clinical phase I trial. Next, Markus Kleinewietfeld, Dresden, reported on the role of NaCl in autoimmunity balancing regulatory T cell (T_reg_) and Th_17_ cell numbers via the p38/MAP kinase pathway and serum- and glucocorticoid-inducible kinase 1 (SGK1). These data indicate a role of dietary habits as environmental factor to modulate autoimmunity most likely via the gut microbiota. Jana Sonner from the group of Michael Platten in Heidelberg gave a presentation on the role of tryptophan catabolites on the development of T_regs_ and Th_17_ cells. A particularly important role of the serine/threonine-protein kinase general control non-derepressible 2 (GCN2) could be shown. Karin Steinbach from the group of Doron Merkler, Geneva, talked about viral footprints in the CNS and their role as a trigger of autoimmunity (fertile field hypothesis). Using the LCMV (non-cytolytic lymphocytic choriomeningitis virus) infection mouse model the group could show that preceeding infection of the brain early in life results in “purged” glial cells that appear to support a higher lesion load following EAE induction.

Next, Alexander Schulz from Jena (research group of Helen Morrison) gave a presentation on neurofibromatosis type II. Merlin, the gene product of NF2, interacts with neuregulin 1, has a strong impact on the myelin sheath, and influences axon structure maintenance. Stefanie Jörg from Ralf Linker’s lab in Erlangen investigated the role of dietary fatty acids and their impact on CNS autoimmunity via the small intestine. The main outcome of the study indicated a differential shift in Th_17_ and Th_1_ cell numbers in the gut induced by lauric acid accompanied by an upregulation in IL17 and IFNg production. In contrast, proprionic acid treatment resulted in upregulated numbers of regulatory T cells associated with an ameliorated disease course in EAE mice.

Marie Wunsch from Stefanie Kuerten’s group in Würzburg presented data on the enteric nervous system as a putative immune target in multiple sclerosis. Focusing on NKG2D, Tobias Ruck from the group of Heinz Wiendl/Sven Meuth (Münster) provided insights in the role of this receptor on T cells in the EAE model as well as in patients suffering from MS or polymyositis. Vivien Thom from the group of Tim Magnus gave a talk on interleukin-17 producing T cells from the CSF as a possible marker for cerebral vasculitis. Christina Ruland from Judith Alferink’s group in Münster talked about tetraspanin-2 as a new murine tetraspanin in peripheral immune cells including neutrophils, CD4 positive T cells, and conventional dendritic cells as well as oligodendrocytes. Lena Krzyzak from Alexander Steinkasserer’s group in Erlangen focused on the role CD83 in the immune response. Membrane bound C83 is present in thymic epithelial cells, dendritic cells, and regulatory T cells exerting different functions. In contrast soluble CD83 has an immunmodulatory role via modulation of IDO.

### Neuroscience and neurodegeneration

Paul Lingor from Göttingen reported on Rho kinase (ROCK) inhibition as a therapeutic target for Amyotrophic Lateral Sclerosis (ALS) and Parkinson’s disease (PD). ROCK inhibition by Fasudil was able to improve functional outcome, reduce astrogliosis and increase numbers of microglial cells in a mouse model of amyotrophic lateral sclerosis (SOD1^G93A^ mice) and showed neuroprotection in PD models.

In a project by Markus Krohn from Magdeburg (group of Jens Pahnke), APPPS1 mice were crossed onto FvB or C57BL/6 backgrounds. Notably, the Ab42 peptide load in the CNS of FVB mice was significantly lower than in the C57BL/6 strain. Conversely, the extent of microglia activation was higher in the FVB background suggesting that more active microglia in FVB mice were responsible for the reduced plaque size in FVB mice. An interesting breeding approach revealed that the differential microglia activation between the strains might be due to mitochondrial differences in the microglia.

Ria Uhlemann from Mathias Endres’ group described how NO release by microglia is modified by a post-transcriptional mechanism involving the actin cytoskeleton. Actin filament stabilization in M1 polarised microglia led to reduced NO production and reduced TNF and IL-6 but increased IL-1b release. Similar results were observed in Gelsolin KO mice. These observations highlight the role for exocytotic pathways in microglial cells as potential targets of interventional strategies in conditions with chronic microglia activation.

Finally, Teja Groemer from the psychiatry department of Erlangen presented new observations on presynaptic APP trafficking. Cleaved APP products, Ab40 and Ab42, are important biomarkers for neurodegenerative diseases, in particular Alzheimer’s disease. APP is not part of synaptic vesicles although its secretion is modulated by synaptic activity. In fact, BACE and APP are sorted away from synaptic vesicles in the presynaptic buton and the “branching points” of these vesicle trafficking pathways are under active investigation.

In conclusion, this year’s NEUROWIND meeting once again proved to be an exciting platform for bringing together highly motivated young scientists in experimental neurology to discuss scientific progress in the fields of neuroimmunology, vascular biology, and neurodegeneration.

